# Ambulatory Orchidopexy Is a Potential Solution to Improve the Rate of Timely Repair in Cryptorchid Boys: An 8 Year Retrospective Study of 4,972 Cases

**DOI:** 10.3389/fped.2021.671578

**Published:** 2021-05-04

**Authors:** Tianxin Zhao, Fuming Deng, Wei Jia, Xiaofeng Gao, Zhongmin Li, Xiangliang Tang, Dian Li, Rui Zhou, Fangpeng Shu, Jin Zhang, Zhengtao Zhang, Wen Fu, Guochang Liu

**Affiliations:** ^1^Department of Pediatric Urology, Guangzhou Women and Children's Medical Center, Guangzhou Medical University, Guangzhou, China; ^2^Department of Pediatric Surgery, Guangzhou Institute of Pediatrics, Guangzhou Women and Children's Medical Center, Guangzhou Medical University, Guangzhou, China

**Keywords:** ambulatory surgery, birth defect, cryptorchidism, orchidopexy, timely repair, medical resources

## Abstract

**Background:** Cryptorchidism is the most common congenital anomaly in pediatric urology. Although early surgery on cryptorchid boys is recommended by pediatric urologists worldwide, the actual age at orchidopexy is often older than the recommended age. Our medical center has started performing ambulatory orchidopexy since March 2016 at the ambulatory surgery center. We aimed to investigate whether ambulatory orchidopexy can improve the timely repair rate.

**Methods:** A retrospective analysis was conducted from 2012 to 2019 at our medical center. Ambulatory orchidopexy was started at our medical center on March 24, 2016. Boys born on or after September 24, 2015 were classified into the “with ambulatory medical resource” group, and boys born before September 24, 2014, were classified into the “without ambulatory medical resource” group. The timely repair rates were calculated and compared.

**Results:** A total of 4,972 cryptorchidism cases were included in the final study. Approximately 33.0% of cryptorchid boys received timely surgery (orchidopexy by the age of 18 months), and only 6.8% of all cryptorchid boys underwent surgery before the age of 1 year. After the performance of ambulatory orchidopexy, the timely repair rate increased from 25.7 to 37.0% (*P* < 0.001), and the percentage of patients receiving surgery before the age of 1 year increased significantly from 3.5 to 8.6% (*P* < 0.001). The proportion of timely repair in patients with ambulatory medical resources was significantly higher than that in patients without ambulatory medical resources (15.6% vs. 58.2%, *P* < 0.001). Significant changes in the rate of surgery before 12 months of age were also found between the two groups (2.4% vs. 14.8%, *P* < 0.001).

**Conclusions:** After the performance of ambulatory orchidopexy in our medical center, the rates of both timely repair and receiving surgery before the age of 1 year increased significantly. Ambulatory orchidopexy is a potential solution to improve the rate of timely repair in cryptorchid boys, and it is worthy of promotion in developing countries and regions.

## Introduction

Cryptorchidism, or undescended testis, is characterized by the absence of testicles in the scrotum, which is the result of failed testicular descent during the fetal period (1). It is the most common congenital anomaly in pediatric urology, occurring in ~1 to 9% of all neonates. In cases of cryptorchidism at birth, testicles of about half of the boys will descend spontaneously within the first 3 months of life; however, about 1% of boys will still have undescended testis at the age of 1 year, and this situation could remain static for years (2–6).

Untreated cryptorchidism is highly associated with impaired fertility (7–10), testicular malignant degeneration (9, 11), testicular torsion (12, 13), and inguinal hernia (14, 15). Orchidopexy, an operation to stabilize the undescended testis in the scrotum, is considered as the standard treatment for cryptorchidism. Studies have reported that early orchidopexy can significantly reduce the risk of adverse consequences of cryptorchidism (1, 11). Therefore, early surgery on cryptorchid boys is recommended by pediatric urologists worldwide (16–18).

The American Urologic Association (AUA) (19) and the European Association of Urology (EAU) (20) recommend that 6-month-old boys with cryptorchidism should undergo surgery within the next 1 year (by 18 months of age). The Nordic consensus suggests that orchidopexy should be performed when patients are in the age range from 6 to 12 months (21). The consensus of Chinese pediatric surgeons recommends that the operation age of cryptorchidism starts at 6 months, preferably before 12 months, and at the latest, before 18 months (22).

However, reports from many countries around the world have shown that the actual age at orchidopexy is often later than the recommended age, and timely repair is not available for most of the patients (23–26). In the United States, Savoie et al. (24) reported that 27% of 1,209 patients with cryptorchidism had surgical correction before 18 months of age, and Kokorowski et al. (27) found that only 18% of the 28204 cryptorchid boys underwent surgery before the age of 1 year. In a German cohort study, Boehme et al. (28) reported that up to 8% of boys with cryptorchidism underwent surgery before the age of 1 year. In Sweden, Bergbrant et al. (29) conducted a nationwide study and reported that 5.9% of patients had surgery before the recommended 1 year of age in 2014. In China, Zhao et al. (26) reported that 16.9% of 2,423 patients received timely repair by 18 months of age.

How to get timely surgical treatment for children with cryptorchidism has attracted widespread attention. Several studies have explored various aspects, such as guidelines intention (28), referral patterns (23), and clinical and socioeconomic factors related to delayed surgery (26, 27, 30), but so far there is a lack of appropriate strategies to further promote early repair of cryptorchidism in children.

Ambulatory surgery, also known as day surgery, was introduced in 1970. This practice has been widely accepted due to the relative benefits to patients and economic savings (31). In 1987, Roth et al. (32) reported their successful experience of ambulatory surgery in cryptorchidism at a local hospital in the United States and found that the procedures were safe, less time-consuming, and economical. Since then, ambulatory orchidopexy has gradually been practiced in many hospitals around the world, while the time to start day surgery of orchidopexy varies in different regions (33–37).

Our medical center, as a regional children's Medical Center in central and southern China, has started performing ambulatory orchidopexy for cryptorchid boys since March 24, 2016. With the development of daytime surgery for cryptorchidism, we have realized that the rate of older children undergoing surgical repair has decreased in recent years. We speculate that performing ambulatory orchidopexy may be a potential solution to improve the rate of timely repair in cryptorchid boys.

Therefore, in this study, we conducted a retrospective analysis of all orchidopexies performed in our center from January 2012 to December 2019, evaluated the proportion of cryptorchid boys receiving timely repair and its trend, and analyzed the association between ambulatory orchidopexy and timely repair rate. This research aims to investigate and share the medical experience of improving the timely repair rate of cryptorchidism from a new perspective.

## Materials and Methods

### Data Collection

**Figure 1:**
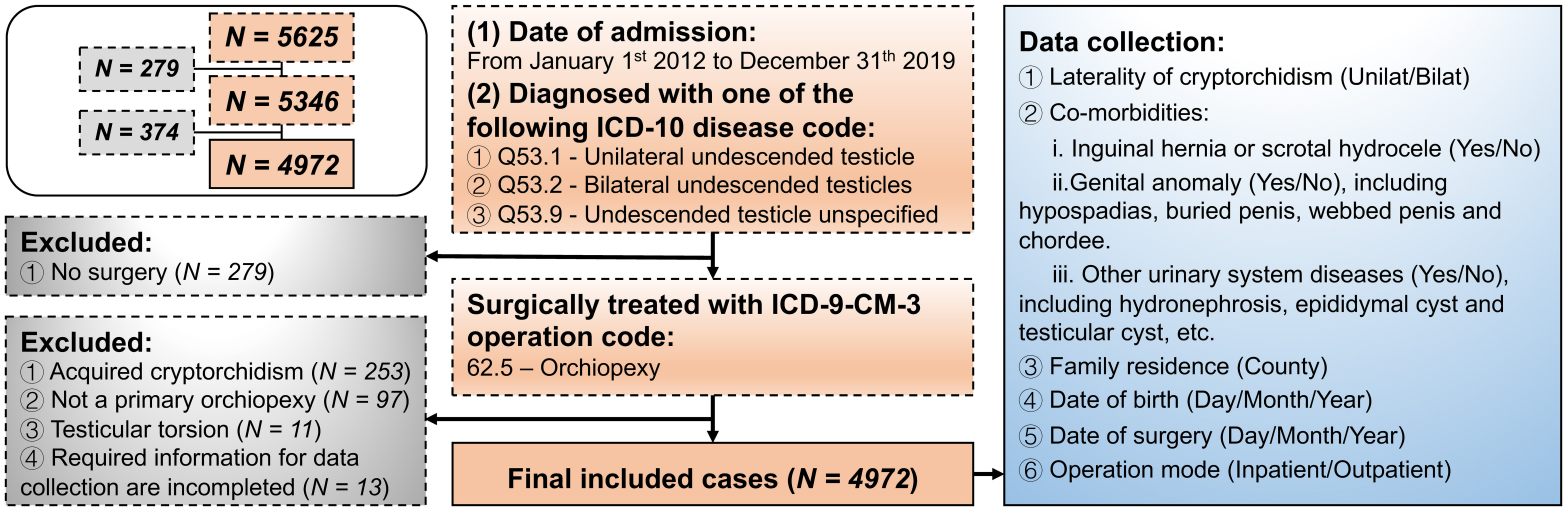
Flow diagram of the retrospective protocol for case inclusion and exclusion and data collection.

A retrospective review was performed on boys who received orchidopexy from January 2012 to December 2019 at Guangzhou Women and Children's Medical Center, the national children's regional medical center in central and south China, after receiving a diagnosis of cryptorchidism. Patients were identified by diagnostic and operative codes for cryptorchidism and orchidopexy from the electronic record system. The process of inclusion and exclusion of study cases is shown in Figure 1.

Data of demographic and clinical information and other details of surgery were extracted. Demographic data included date of birth and county where the patients lived. Whether the patients lived in a poor county or not was defined according to the list of poor counties in China (http://www.cpad.gov.cn/art/2012/3/19/art_50_23706.html, accessed on 10th September 2020), which was determined based on the average annual income of the local population. With respect to clinical information, data on the side affected by cryptorchidism and comorbidities were collected. Age at orchidopexy was calculated from the date of birth to the date of surgery. Date of surgery and operation mode (hospitalized or ambulatory orchidopexy) were also obtained.

### Hospitalized and Ambulatory Orchidopexies

For hospitalized orchidopexy, patients needed to take an appointment for bed in advance, undergo complete preoperative examination during hospitalization, receive surgical treatment, and recover after the operation until discharge indication was reached, including normal body temperature, no exudation from the wound, and eating easily without any abdominal distension in case of laparoscopic surgery. For ambulatory orchidopexy, the patients completed preoperative examination before admission, received surgical treatment, underwent short-term observation, and achieved discharge within 4–12 h in the ambulatory surgery center. Specially, enough postoperative observation time (at least 8 h) is guaranteed in case of laparoscopic orchidopexy. Ambulatory orchidopexy was performed by an experienced pediatric urologist, and anesthesia management was strictly carried out by a pediatric anesthesiologist. If postoperative complications occurred after discharge, the pediatric surgery emergency department of our hospital was available and the chief resident of pediatric urology was on full duty.

The choice of operation mode was made based on the AUA guideline (19) and the consensus among Chinese pediatric urologists (22). Briefly, open surgery, including the inguinal approach and trans-scrotal approach, was used for palpable testis, and trans-scrotal orchidopexy was especially used for undescended testes distal to the external inguinal ring. Laparoscopic surgery was used for impalpable testis.

### Group Setting

According to the recommendation in the AUA and EAU guidelines suggesting that surgical repair for cryptorchidism should be performed by 18 months of age (19, 20), we defined timely repair as primary orchidopexy performed before the age of 18 months. Based on that, our medical center started performing ambulatory orchidopexy since March 24, 2016. For boys born on or after September 24, 2015, if they were diagnosed with cryptorchidism at the age of 6 months and determined to receive surgical repair, they had the medical resource of ambulatory orchidopexy. Thus, they were classified as the “with ambulatory medical resource” group. For boys born before September 24, 2014, they did not have the medical resource of ambulatory surgery until the latest time for timely surgery (18 months of age); hence, they were classified as the “without ambulatory medical resource” group.

### Statistical Analyses

Statistical analyses were conducted with SPSS version 23.0 (SPSS Inc., Chicago, IL, USA). Dichotomous variables were compared using Chi-squared tests. Normally and non-normally distributed continuous variables were compared by the Student's *t*-test and Mann-Whitney *U* test, respectively. Variables, which were determined to be significant in the univariate analyses, were included in the binary logistic regression analysis. Data were considered significant at *P* < 0.05.

## Results

A total of 4,972 patients receiving orchidopexy in Guangzhou Women and Children's Medical Center were included in this study (Table 1). The median age at orchidopexy was 23 months. A total of 40.7% of cryptorchid boys underwent ambulatory surgery. In most patients, cryptorchidism was unilateral (84.8%). Nearly 10.3% of patients had inguinal hernia or scrotal hydrocele. A few cryptorchidism cases had accompanying genital anomaly (2.7%) and other urinary system diseases (1.6%). About 2.6% of patients lived in a poor county.

**Table 1:** Demographic, clinical, and socioeconomic characteristics of 4,972 cases.

	**Total** **(*n* = 4,972)**	**Timely repair** **(*n* = 1,642)**	**Delayed repair** **(*n* = 3,330)**	***P***
Age at orchidopexy (months), median (IQR)	23 (27)	14 (4)	33 (36)	<0.001
Average age (months), mean ± SD	35.4 ± 30.0	14.1 ± 2.4	45.9 ± 31.7	<0.001
Ambulatory orchidopexy, No. (%)	2,025 (40.7)	776 (47.3)	1,249 (37.5)	<0.001
Unilateral cryptorchidism, No. (%)	4,218 (84.8)	1,416 (86.2)	2,802 (84.1)	0.053
Comorbidity, No. (%)				
IH/SH	514 (10.3)	172 (10.5)	342 (10.3)	0.824
Genital anomaly	167 (2.7)	44 (3.7)	123 (3.4)	0.062
Other urinary diseases	82 (1.6)	25 (1.5)	57 (1.7)	0.622
Poverty-stricken county residence, No. (%)	127 (2.6)	25 (1.5)	102 (3.1)	0.001

*IH, inguinal hernia; SH, scrotal hydrocele; IQR, interquartile range; SD, standard deviation*.

The above demographic, clinical, and socioeconomic factors in the timely repair group and delayed repair group were compared in univariate analysis (Table 1). There was a significant difference only in living in a poor county or not between these patients (2.6% vs. 1.5%, *P* < 0.001). Then, this factor was included in a logistic regression analysis, and the result showed that boys with cryptorchidism who lived in a poor county were at a high risk of undergoing delayed repair (OR = 2.047, 95% CI: 1.316, 3.183) (Supplementary Table 1).

**Figure 2:**
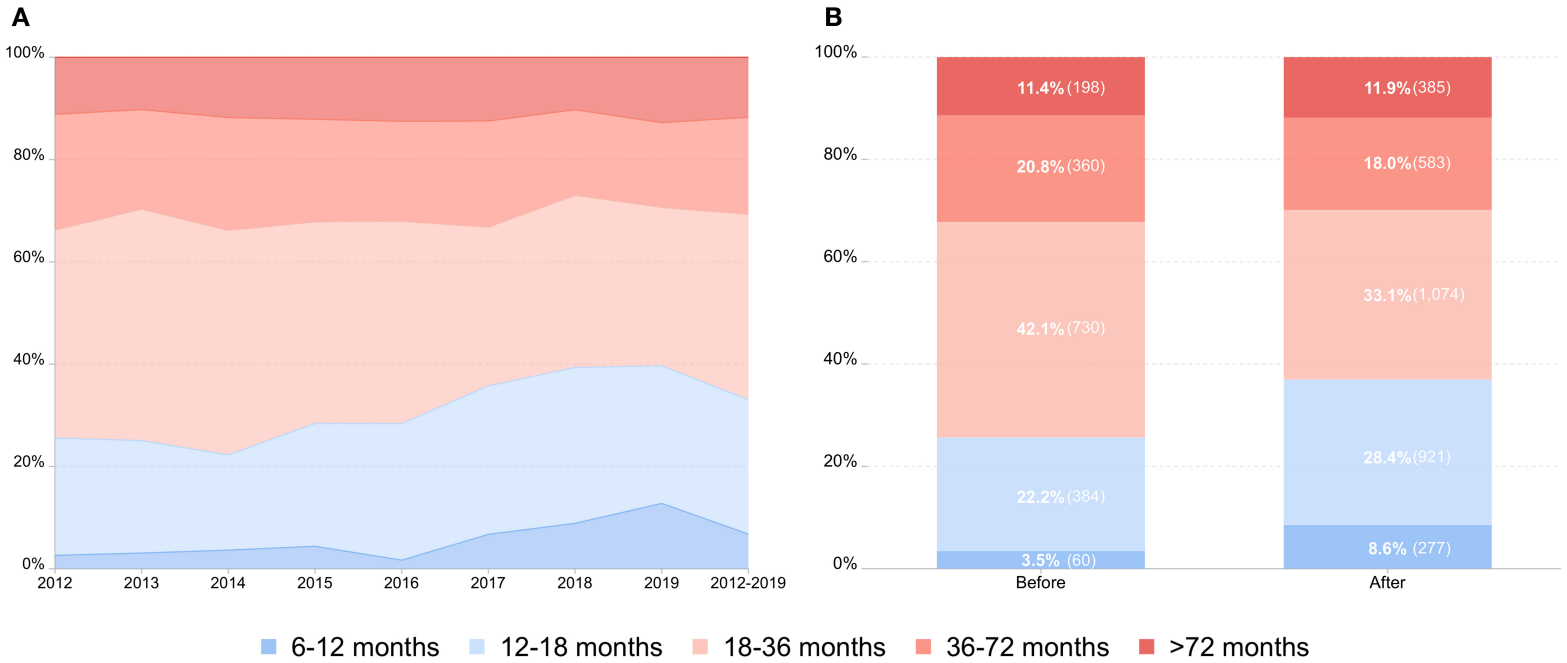
Age distribution and comparison of the repair time in cryptorchid boys. **(A)** Age distribution of children with cryptorchidism undergoing primary orchidopexy from 2012 to 2019 by the age category. **(B)** Comparison of the repair time in cryptorchid boys before and after the hospital performed ambulatory orchidopexy.

Overall, 1,642 patients (33.0%) received timely surgical repair and underwent orchidopexies by 18 months of age. Further, only 337 patients (6.8%) underwent surgery before the age of 1 year. On and before 2016, the proportion of timely repair ranged from 22.3 to 28.4%. From 2017 to 2019, the proportion of timely repair ranged from 35.8 to 39.7% (Figure 2A).

Our medical center has been performing ambulatory orchidopexy since March 24, 2016. Before (from January 1, 2012 to March 23, 2016) and after (from March 24, 2016 to December 31, 2019) the performance of ambulatory surgery, 1,732 (34.8%) and 3,240 (65.2%) patients were operated upon, respectively. There was no significant difference between the two groups before and after ambulatory surgery (3.1% vs. 2.3%, *P* = 0.098) (Supplementary Table 2). Before the day surgery of orchidopexy, 444 patients (25.7%) received timely repair, and 60 patients (3.5%) underwent surgery before the age of 1 year. After the performance of ambulatory surgery, 1,198 patients (37.0%) received timely repair and the number of patients undergoing surgery before the age of 1 year increased to 277 patients (8.6%) (Figure 2B).

**Figure 3:**
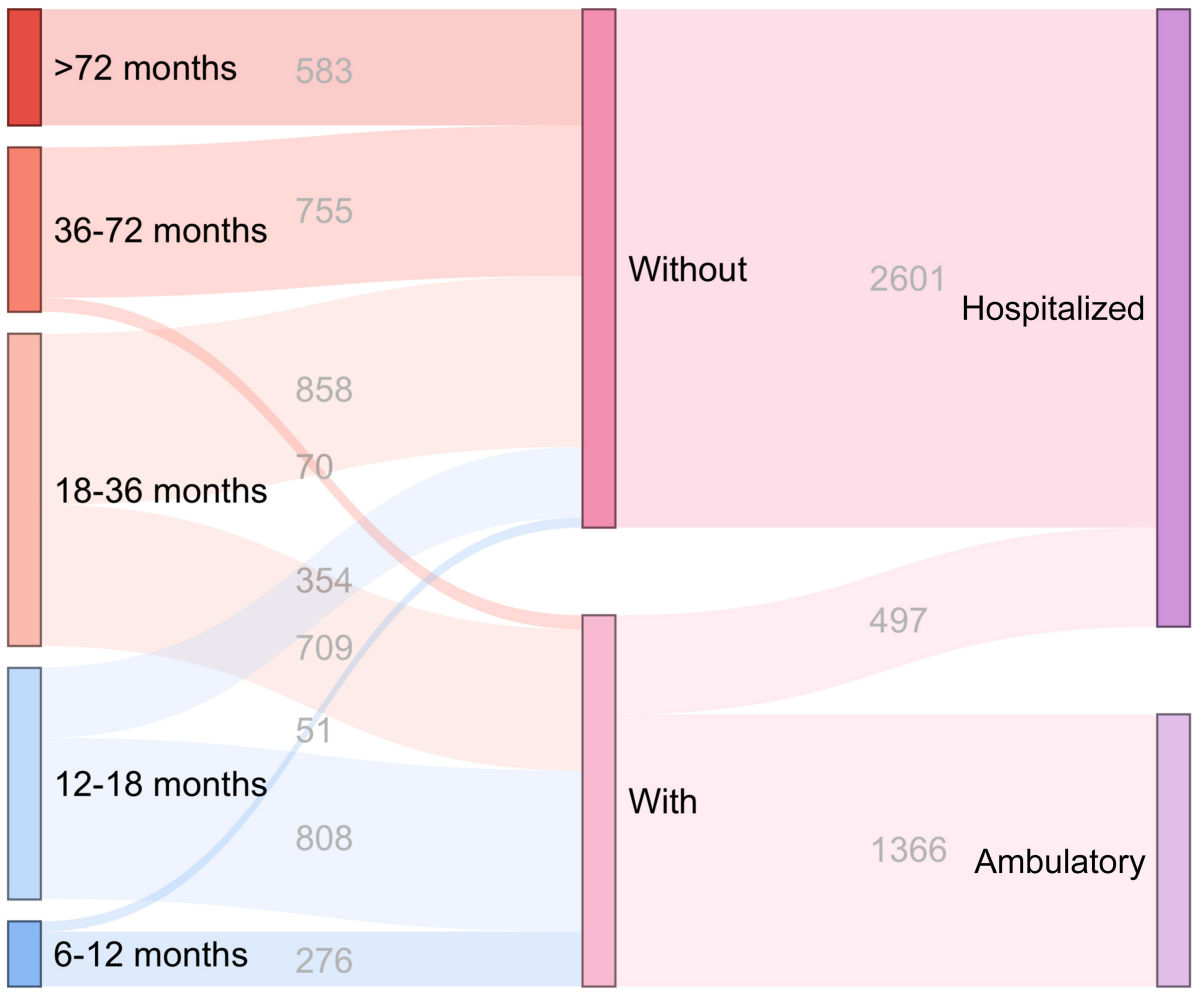
Distribution of the repair time and operation mode in cryptorchid boys with and without the medical resource of ambulatory orchidopexy. “6–12 months,” “12–18 months,” “18–36 months,” “36–72 months,” and “>72 months” indicate the repair time in cryptorchid boys. “With” and “Without” indicate the cryptorchid boys with and without the medical resource of ambulatory orchidopexy, respectively. “Hospitalized” indicates the cryptorchid boys undergoing hospitalized orchidopexy, and “Ambulatory” indicates the cryptorchid boys undergoing ambulatory orchidopexy.

There were 1,863 boys in the “with ambulatory medical resource” group and 2,601 boys in the “without ambulatory medical resource” group. No significant difference was found between the two groups with and without ambulatory medical resources (3.0% vs. 2.1%, *P* = 0.085) (Supplementary Table 2). The proportion of timely repair in patients with ambulatory medical resources was significantly higher than that in patients in the “without ambulatory medical resource” group (15.6% vs. 58.2%, *P* < 0.001). Significant changes in the rate of surgery before 12 months of age were also found between the two groups (2.4% vs. 14.8%, *P* < 0.001). Of all patients in the “with ambulatory medical resource” group, 1,366 patients (73.3%) received ambulatory orchidopexy (Figure 3).

## Discussion

Pediatric urologists used to evaluate ambulatory orchidopexy, mostly focusing on the safety and effect of health economics. It is well-documented that ambulatory surgery for cryptorchidism is reliable with a high success rate and few postoperative complications, and reduced medical expenses (33, 34, 38). However, primary cryptorchidism is a special disease in pediatric surgery, as the location of undescended testicles in the groin or abdominal cavity may lead to a series of adverse consequences (1, 11). Therefore, it has been recognized by pediatric urologists worldwide that children with cryptorchidism need early surgery. At present, the medical resources of pediatric surgery in China are not sufficient, and there is still a shortage of pediatric urology specialists. Whether ambulatory orchidopexy can help to improve the timely repair rate remains to be studied.

In our study, we determined 18 months as the latest suitable age for orchidopexy in accordance with the AUA and EAU guidelines. Overall, 33% of patients underwent orchidopexies by 18 months of age from 2012 to 2019. In particular, after the performance of ambulatory orchidopexy, the timely repair rate increased from 25.7 to 37.0%, and the percentage of patients receiving surgery before 1 year increased significantly from 3.5 to 8.6%.

A study at a tertiary pediatric medical center in Southwest China, where ambulatory surgery for cryptorchidism was not regularly performed, reported that 16.9% of children with cryptorchidism received surgical repair before 18 months of age from 2012 to 2017 (26). The timely repair rate in our center is higher than that in the pediatric medical institution in Southwest China. Also, after the performance of ambulatory orchidopexy in our center, the difference in the rates of timely surgery between the two hospitals became even larger. This may also be related to the different levels of economic development in these two places.

A recent study based on the largest German cohort reported that about 8% of cryptorchid boys received surgery before the age of 12 months from 2013 to 2016 (28). This percentage is similar to the proportion observed in our center after the performance of ambulatory surgery. Additionally, there are reports from other regions suggesting that the rate of surgery before 12 months of age ranges from 18 to 21% (27, 39). The rate of early surgery for cryptorchidism found in our study population is still low by international standards, and more work needs to be done to further improve the early operation rate.

We further divided the children with cryptorchidism into the following two groups according to their birth time: “with the medical resource of ambulatory orchidopexy” and “without the medical resource of ambulatory orchidopexy.” Then, we focused on the age of the two groups of children receiving orchidopexy. The results showed that the percentages of timely repair and receiving surgery before the age of 12 months in boys with medical resources of ambulatory orchidopexy were much higher than those in boys without such medical resources. More than half of the boys with medical resources received timely repair, and 14.8% of them received surgery before the age of 12 months. These rates are close to the high rate of early surgery reported internationally (27, 39).

According to our experience, before the ambulatory surgery, due to limited beds in the ward, after the diagnosis of cryptorchidism and confirmation of the need for surgical repair, children often cannot receive surgical treatment in a short period of time because of the waiting period for beds. Alsowayan et al. (40) have reported a similar situation, in which the median waiting time for elective surgery of cryptorchidism was 4.8 months and the long waiting time was related to delayed repair. However, after the performance of ambulatory orchidopexy in our center, due to the sufficient number of daily ambulatory surgeries, children with cryptorchidism can immediately take an appointment for near-term surgery after diagnosis. Our results suggest that hospitals should provide full opportunities for performing ambulatory orchidopexy, which could be an effective solution to improve the rate of timely repair in cryptorchid boys.

Therefore, we summarized the experience of our center in performing ambulatory orchidopexy. First of all, for the hospital management, the ambulatory surgery center should arrange enough operation days for the pediatric urology department to ensure that cryptorchid boys have sufficient appointment opportunities. Second, for the department management, experienced senior doctors should be arranged to perform ambulatory orchidopexy to ensure the surgical treatment effect. Third, for the operation process, hemostasis should be fully achieved during the operation, and special attention should be paid to the details of the operation, such as the position of fixing the testis and suture of the incision, to avoid complications as far as possible.

In this study, we also analyzed the factors related to delayed surgery to make the comparisons of timely repair rates rigorous, and we found that children living in poor counties had a higher risk of delayed repair, which is consistent with a previous report (26). This implies that the level of local economic development would affect children's access to medical care. Although the proportion of the population living in poverty-stricken counties in the present study was low, there was no significant difference in this index between the groups before and after the performance of ambulatory orchidopexy and among children with and without medical resources for ambulatory orchidopexy. This suggests that the comparison results of timely repair ratio between the above two groups is reliable and scientific.

Interestingly, our study found that there was no significant difference in all comorbidities between the timely repair group and delayed repair group, which is different from the previous study. Zhao et al. (26) have reported that the combination of other diseases related to the abnormal appearance of external genitalia may motivate parents to bring their children to urology specialty for care and help in the early diagnosis of cryptorchidism. In this study, the hospital was located in southern China, which has relatively abundant medical resources, and the public's awareness of the disease may be higher. For some children, early attention should be paid by their parents or guardians and they should receive timely treatment even in the absence of appearance abnormalities of the external genitalia. In addition, it has been recognized that early diagnosis is an important factor for encouraging cryptorchid children to undergo surgery at an optimal age. Thus, the primary care providers should palpate the testes for quality and position at each recommended child visit.

The present study had several limitations. First, as it was a retrospective study, the information that this study could collect was limited; thus, it was not able to analyze the surgical effect, postoperative complications, and health economic indicators in detail. Future studies can design prospective research programs, combined with health economics, to explore the effects of ambulatory orchidopexy in promoting early surgery in children with cryptorchidism from many aspects. Second, although our hospital is a regional children's Medical Center in China, the single center study could only reflect the impact of ambulatory orchidopexy on the timing of surgery in children with cryptorchidism in local areas. Next, we will work with regional children's medical centers in other regions of China to evaluate the role of ambulatory orchidopexy in promoting early surgery of cryptorchidism in children under the current medical model in China through multi-center research. Third, the research span of children “with the medical resource of ambulatory orchidopexy” in this study was only 3 years. The current estimated rate of timely surgery in this group may be higher than the actual rate because there still may be some children who did not receive treatment within the study period, and more studies are needed in the future.

## Conclusions

Approximately 33.0% of cryptorchid boys in our study received timely surgery (orchidopexy by the age of 18 months), and only 6.8% of all boys underwent surgery before the age of 1 year. After the performance of ambulatory orchidopexy in our medical center, the rates of both timely repair and receiving surgery before 1 year increased significantly. These proportions in cryptorchid boys with ambulatory medical resource were significantly higher than those in boys without ambulatory medical resource. To improve the rate of timely repair in cryptorchid boys, promoting ambulatory orchidopexy could be a potential solution.

## Data Availability Statement

The raw data supporting the conclusions of this article will be made available by the authors, without undue reservation.

## Author Contributions

TZ designed the study, carried out data analysis, and drafted the manuscript. FD and WJ participated in data analysis and helped in drafting the manuscript. XG, ZL, XT, DL, RZ, FS, JZ, and ZZ participated in data analysis, collected all relevant data, and assisted in study conception and design. WF and GL conceived the study, participated in its design and coordination, and helped in drafting the manuscript. All authors read and approved the final manuscript.

## Conflict of Interest

The authors declare that the research was conducted in the absence of any commercial or financial relationships that could be construed as a potential conflict of interest.
