# Supplementary material for: Ambulatory Orchidopexy Is a Potential Solution to Improve the Rate of Timely Repair in Cryptorchid Boys: An 8 Year Retrospective Study of 4,972 Cases
Source: Front Pediatr. 2021 May 4;9:671578. doi: 10.3389/fped.2021.671578 (PMC8129512; doi:10.3389/fped.2021.671578)
Supplement: Supplementary file 1 [file Table_1.docx]

**Supplementary Tables**

**Supplemental Table 1. Logistic regression analysis of the factor associated with delayed surgical repair**

**Supplemental Table 2. Distribution of poverty-stricken county residences under different grouping conditions**

**Supplemental Table 1: Logistic regression analysis of the factor associated with delayed surgical repair**

|  | Parameter estimate | SE | *P* | OR (95% CI) |
| --- | --- | --- | --- | --- |
| Living in a poverty-stricken county | 0.716 | 0.225 | 0.001 | 2.047 (1.316, 3.183) |

Abbreviations: SE, standard error; OR, odds ratio; CI, confidence interval

**Supplemental Table 2: Distribution of poverty-stricken county residences under different grouping conditions**

|  | Poverty-stricken county residence, No. (%) | *P* |
| --- | --- | --- |
| Before/after the hospital could perform ambulatory orchidopexy^a^ |  | 0.098 |
| Before (n=1732) | 53 (3.1) |  |
| After (n=3240) | 74 (2.3) |  |
| Cryptorchid boys with/without medical resources for ambulatory orchidopexy |  | 0.085 |
| Without^b^ (n=2601) | 76 (2.9) |  |
| With^c^ (n=1863) | 39 (2.1) |  |

^a^The hospital started ambulatory orchidopexy on March 24, 2016.

^b^Defined as born before September 24, 2014.

^c^Defined as born on or after September 24, 2015.
